# [^2^H_26_]-1-*epi*-Cubenol, a completely deuterated natural product from *Streptomyces griseus*

**DOI:** 10.3762/bjoc.9.319

**Published:** 2013-12-10

**Authors:** Christian A Citron, Jeroen S Dickschat

**Affiliations:** 1 Institut für Organische Chemie, Technische Universität Braunschweig, Hagenring 30, 38106 Braunschweig, Germany

**Keywords:** biosynthesis, deuterium, labelling, natural products, *Streptomyces*, terpenes

## Abstract

During growth on fully deuterated medium the volatile terpene [^2^H_26_]-1-*epi*-cubenol was released by the actinomycete *Streptomyces griseus*. This compound represents the first completely deuterated terpene obtained by fermentation. Despite a few previous reports in the literature the operability of this approach to fully deuterated compounds is still surprising, because the strong kinetic isotope effect of deuterium is known to slow down all metabolic processes in living organisms. Potential applications of completely labelled compounds from natural sources in structure elucidation, biosynthetic or pharmacokinetic investigations are discussed.

## Introduction

**Figure 1:**
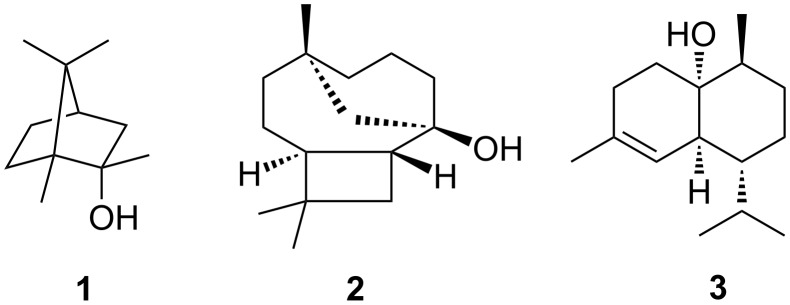
Terpenoids from *Streptomyces griseus*.

The actinomycete *Streptomyces griseus* is a producer of three terpenes, 2-methylisoborneol (2-MIB, **1**), (+)-caryolan-1-ol (**2**), and (+)-1-*epi*-cubenol (**3**, Figure 1). The biosynthesis of **2** and **3** requires the action of well characterized terpene cyclases [1–2], while the biosynthesis of the homomonoterpene **1** proceeds by *S*-adenosylmethionine-dependent methylation of GPP followed by a cyclisation reaction [3–5]. For biosynthetic studies on secondary metabolites isotopically labelled precursors are frequently used. Historically, the usage of radiolabelled compounds was most important, because incorporation can be detected with high sensitivity by scintillation counting or autoradiography. However, the site of incorporation was in most cases difficult to deduce, requiring a complex and laborious chemical degradation that was sometimes even performed without knowledge of the full structure of the natural product [6]. A drawback is the health risk emanating from radioactively labelled compounds, thus requiring high safety standards in their handling. Feeding experiments are today in most cases performed with stable isotopes [7]. For ^13^C-labelled compounds the locus of incorporation can be detected by ^13^C NMR spectroscopy. As we have recently shown the sensitivity of modern ^13^C NMR spectrometers is even good enough for analysis of microgram amounts of isotopically enriched material in complex headspace extracts [8–9]. Deuterium is very powerful, particularly when hydrogen shifts [10], stereospecific conversions along multistep biosynthetic pathways [11], hydride abstractions in oxidation steps [12], or deprotonations [13] are to be followed. Its incorporation is best observed by GC–MS, and if fragmentation mechanisms are known [14], the site of incorporation can be deduced from the mass spectrum. Due to the significantly shorter C–D bond compared to a C–H bond resulting in a lower polarisability and weaker interaction of the deuterated analyte with the stationary phase deuterated compounds can be gas chromatographically separated from their non-labelled counterparts [15], allowing for a detailed interpretation of the mass spectrum of a deuterated analyte. A drawback is that high incorporation rates are required to investigate the site of incorporation by ^1^H NMR spectroscopy, but ^2^H NMR spectroscopy offers a suitable alternative [16–17]. Furthermore, deuterium kinetic isotope effects [18] may alter product distributions in branching biosynthetic pathways, which can be useful for their mechanistic interpretation [19]. At high deuterium concentrations such kinetic isotope effects slow down all metabolic processes, and consequently only very few reports on the cultivation of microorganisms in highly deuterated media are available from the literature [20–23]. For eukaryotes high deuterium oxide concentrations are even toxic, e.g., in mice intoxication with deuterium oxide resulted in degenerative defects on multiple organs [24]. Our current research on terpene biosynthesis using stable isotope labellings prompted us to investigate whether it is possible to obtain a fully deuterated terpene by culturing a bacterium in a 100% deuterated medium. Here we report the successful production of the first completely deuterated terpene by fermentation and discuss potential applications.

## Results and Discussion

**Figure 2:**
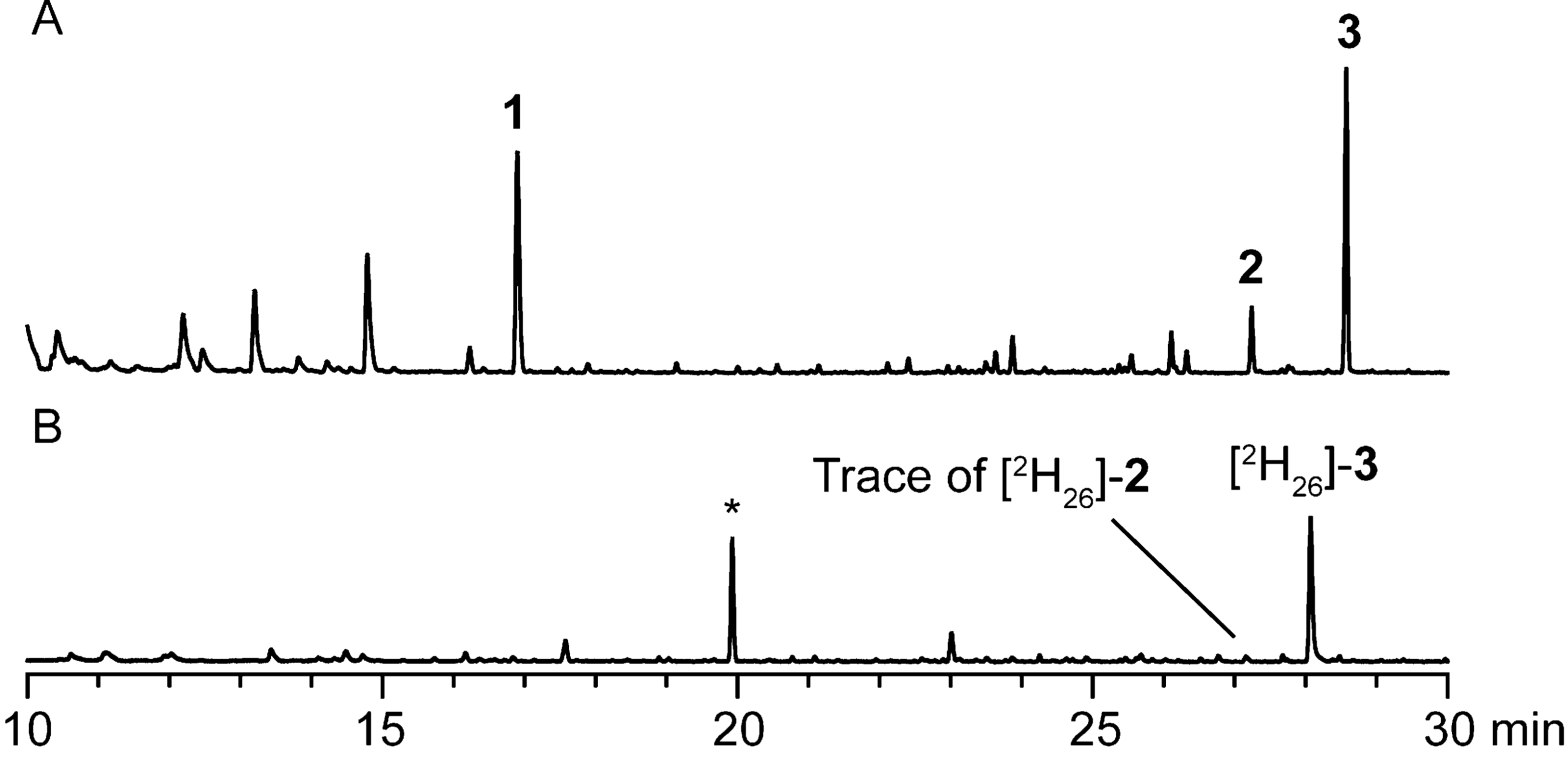
Total ion chromatograms of CLSA headspace extracts from *S. griseus* obtained after (A) incubation on 65.GYM agar and (B) incubation on DMM agar. The peak labelled “[^2^H_26_]-**3**” is composed of [^2^H_26_]-**3** (17%), [^2^H_25_]-**3** (50%), and [^2^H_24_]-**3** (33%), as discussed in detail below. The peak labelled with an asterisk represents a contamination with *exo*-bornyl acetate (non-deuterated) of unknown origin. Deuterated **1** could not be detected.

*Streptomyces griseus* was grown on agar plates containing completely deuterated minimal medium (DMM, ca. 99% deuterium) or normal 65.GYM. While the agar plate cultures on normal 65.GYM were fully grown within two days, the cultures grown on DMM agar required eight to ten days of incubation until fully grown, reflecting the significantly slowed metabolism due to a strong deuterium kinetic isotope effect. After full growth of the culture the volatiles emitted by *S. griseus* were collected on charcoal traps by use of a closed-loop stripping apparatus (CLSA) [25], followed by extraction with dichloromethane and GC–MS analysis of headspace extracts. While 2-MIB (**1**), caryolan-1-ol (**2**) and 1-*epi*-cubenol (**3**) were all produced during growth on full medium (65.GYM), consistent with previous results using other full media [26], only for **3** similar levels of production were observed on DMM (Figure 2). This demonstrated that all relevant terpene biosynthetic pathways including the deoxyxylulose phosphate pathway for the biosynthesis of the terpene monomers dimethylallyl diphosphate (DMAPP) and isopentenyl diphosphate (IPP) as well as polyisoprenoid biosynthesis for the linear precursors geranyl diphosphate (GPP) and farnesyl diphosphate (FPP) were active. However, the volatile **2** was found only in traces, while the production of **1** was completely abolished. Reasons for these findings may be that the production of **2** is under positive control of the bacterial hormone A-factor [1], and A-factor biosynthesis may be significantly delayed on DMM. It is unknown whether the biosynthesis of **1** is also A-factor controlled in *S. griseus*, but an alternative explanation for its abolished production is that the biosynthesis of **1** requires additional chemistry, i.e., a methylation step [3–5]. It is a well described phenomenon that biosynthetic pathways may be differentially expressed under variant culture conditions [27], and the respective pathway to **1** may be downregulated on DMM either due to the different carbon source in this medium or due to a generally slowed down metabolism because of the high deuterium content.

**Figure 3:**
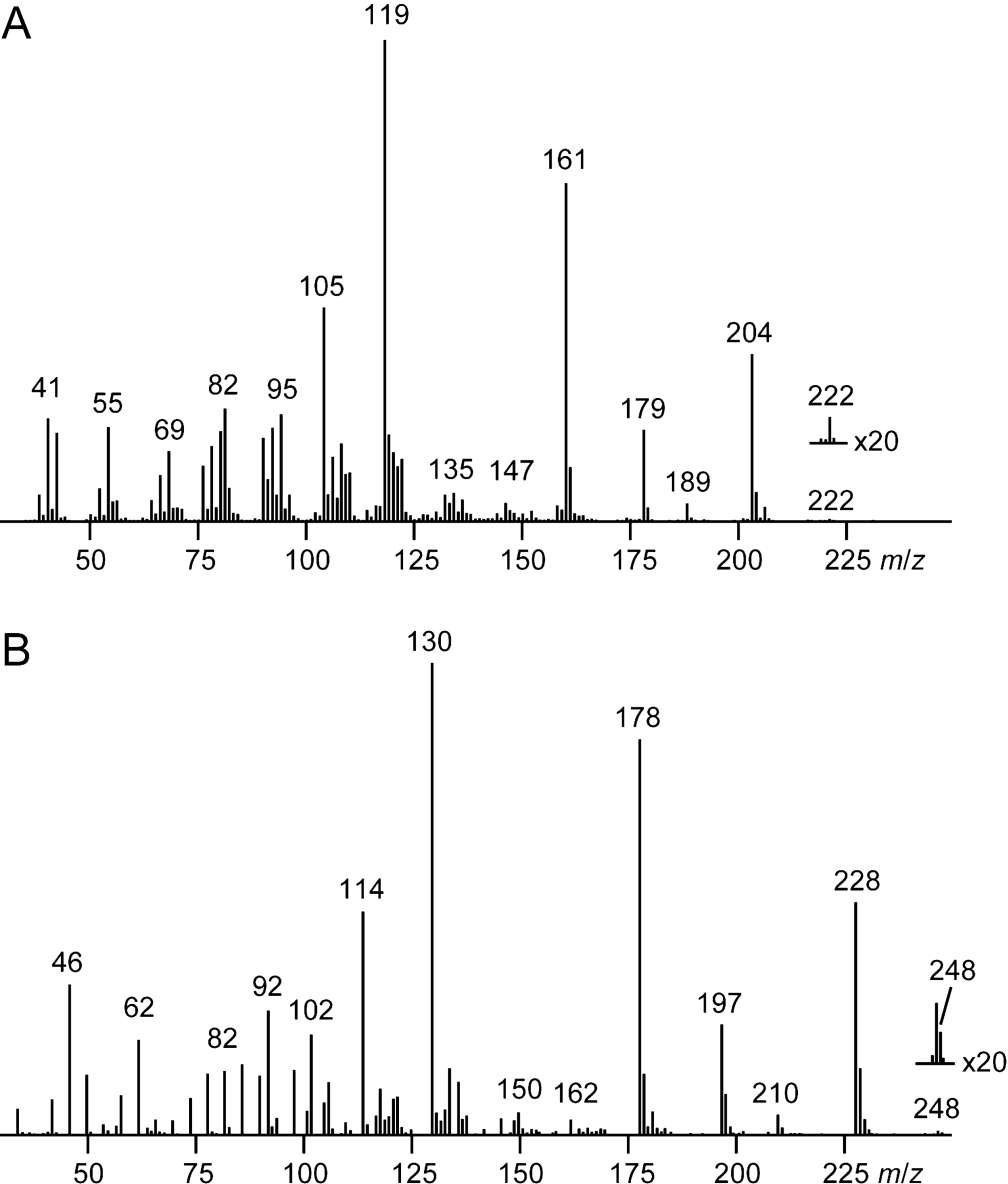
Mass spectra of 1-*epi*-cubenol. (A) Mass spectrum of natural **3** obtained after growth on 65.GYM; (B) mass spectrum of [^2^H_26_]-**3** obtained after growth on DMM.

**Figure 4:**
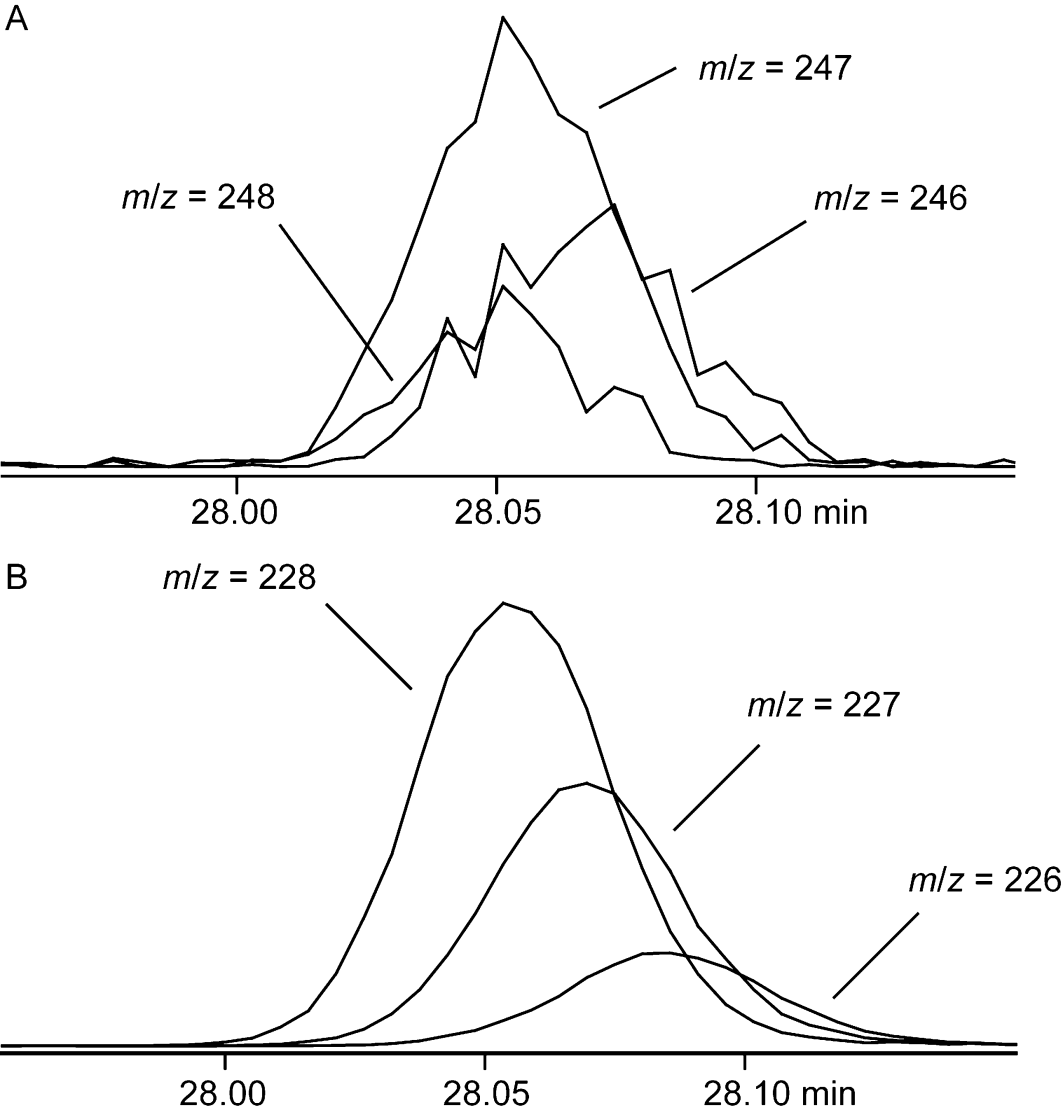
Ion chromatograms of fully deuterated **3** for (A) the molecular ion (*m*/*z* = 248, 247, and 246), and (B) the fragment ion arising by neutral loss of water (*m*/*z* = 228, 227, and 226).

Deuterated **3** eluted almost 1 min earlier than unlabeled *epi*-cubenol. Its mass spectrum obtained after growth on DMM in comparison to the natural compound revealed a shifted molecular ion from *m*/*z* = 222 for the sesquiterpene alcohol **3** (C_15_H_26_0) to *m*/*z* = 248 for deuterated **3** (Figure 3), clearly indicating the uptake of up to 26 deuterium atoms. A detailed analysis of the ion chromatograms of the ions *m*/*z* = 248, 247, and 246 (Figure 4A) demonstrated that 17% of the material were completely deuterated, while 50% of the material represented [^2^H_25_]-**3** and 33% were [^2^H_24_]-**3**. Analysis of the corresponding ion traces of the fragment ions formed by neutral loss of water (*m*/*z* = 228, 227, and 226, Figure 4B) showed that formation of these ions is mainly due to loss of HOD from [^2^H_25_]-**3** and, secondly, of D_2_O from [^2^H_26_]-**3**, resulting in an abundance of the maximum deuterated fragment ion at *m*/*z* = 228 of 55%. Conclusively, the main content of ^1^H resides in the alcohol function of deuterated **3** that is explained by a D–H exchange during CLSA sampling, workup of the sample, or during chromatography that can result from any contact with traces of moisture. At the same time this detailed analysis of ion chromatograms revealed that the carbon backbone of **3** is completely deuterated (only ca. 2% of the whole hydrogen content in **3** were ^1^H). The major fragment ions of **3** at *m*/*z* = 204, 179, 161, 119, and 105 gave clear shifts to *m*/*z* = 228, 197, 178, 130, and 114, much clearer as in only partially deuterated compounds where H,D-scrambling along the carbon backbone is usually observed in mass spectrometric analyses. The advantage of these clear mass shifts is that the deuterium content of each fragment ion can immediately be deduced, e.g., the shift of the base peak ion from *m*/*z* = 119 to 130 points to eleven deuterium atoms. This in turn directly reveals the number of hydrogens in each of the fragment ions also for the unlabelled analyte, without the need for high resolution mass spectrometry (HRMS), similar to a recent report using ^13^C and ^15^N labellings for the determination of the numbers of carbons and nitrogens in a natural product [28]. Therefore, such labelling experiments using a growth medium with a deuterium content of nearly 100% offer a suitable alternative to determine the number of hydrogens in an analyte, if no HRMS instrument is available. As pointed out in Bode’s study a labelling approach may even be more accurate for determination of the sum formula of a natural product, because a measured accurate mass may be in agreement with more than one molecular composition [28].

Completely deuterated compounds as exemplified by [^2^H_26_]-*epi*-cubenol in this work are not only a curiosity, but are also of interest to natural product chemists for the elucidation of biosynthetic pathways. Interesting options for their applications in biosynthetic studies include the use of non-labelled compounds in an inverse feeding experiment [29–31]. Such inverse feedings have a much broader scope, because many more non-labelled compounds than labelled precursors are commercially available. Deuterated compounds can also be applied in pharmacokinetic investigations by tracing the isotopic label in the catabolites of a pharmacological agent [32]. Another potential application of uniformly deuterated compounds is in neutron crystallography that takes advantage of the enhanced visibility of deuterium as compared to hydrogen [33]. Generally, deuterated compounds can synthetically be obtained by hydrogen isotope exchange reactions using Lewis acid, transition metal or enzyme catalysis [34], but such approaches may not be suitable for terpenes that are in many cases unstable under acidic conditions or may tend to thermal rearrangements [35]. The successful formation of a completely deuterated natural product (up to 90% ^2^H) by fermentation has recently been reported for rhamnolipids in *Pseudomonas* and sophorolipids in *Candida apicola* [36], but no examples for completely deuterated terpenes with a deuterium content of >98% as presented here are known. In conclusion, fermentation in fully deuterated medium as performed in this work may offer a good and practicable approach to many other structurally complex and completely deuterated natural products that are difficult to access by synthetic methods.

## Conclusion

In summary, we have observed the first nearly 100% deuterated terpene in bacterial headspace extracts, and discussed the various potential applications of completely deuterated compounds for structure elucidations, biosynthetic or pharmacokinetic investigations that reach far beyond the initially curiosity-driven starting point of this work.

## Experimental

**Strain and culture conditions.** The actinomycete *Streptomyces griseus* NBRC102592 was obtained from the National Biological Research Center, Chiba, Japan. Medium ingredients and dichloromethane (GC grade) were purchased from Carl Roth GmbH (Karlsruhe, Germany). Incubation was performed on regular (non-labelled) 65.GYM agar (glucose: 4.0 g, yeast extract: 4.0 g, malt extract: 10.0 g, CaCO_3_: 2.0 g, agar: 12.0 g, H_2_O: 1000 mL) for a control of terpene production under normal growth conditions, or fully deuterated minimal medium (DMM, [^2^H_8_]glycerol: 5 mL, (N^2^H_4_)SO_4_: 1.0 g, K_2_[^2^H]PO_4_: 0.5 g, MgSO_4_·7[^2^H_2_]O: 0.2 g, FeSO_4_·7[^2^H_2_]O: 0.01 g, agar: 12.0 g, [^2^H_2_]O: 1000 mL) to obtain [^2^H_26_]-**3**. DMM liquid culture medium was devoid of agar and CaCO_3_. Deuterated [^2^H_8_]glycerol (99% ^2^H) was obtained from Eurisotop (Saarbrücken, Germany), deuterium oxide (99.9% ^2^H) was obtained from Deutero GmbH (Kastellaun, Germany), and all deuterated hydrated salts were obtained by dissolving the non-deuterated analogs in a large excess of deuterium oxide (100 equiv) followed by lyophilization. Agar that is composed of ca. 1 wt % of hydrogen atoms that are exchangable with water was added as the only unlabelled compound. The added agar (12 g L^−1^) is equal to ca. 1 mL of water (0.1% of the medium).

**Headspace analysis.** For analysis of the volatiles liquid cultures were inoculated and incubated at 28 °C for 2 d (65.GYM) or 8–10 d (DMM). From these cultures 1 mL was transferred onto agar plates containing the same medium, and incubation was continued for 2 d (65.GYM) or 7–10 d (DMM). The volatile products emitted by the agar plate cultures were collected by use of a closed-loop stripping apparatus (CLSA) [25]. Briefly, in a closed apparatus containing the agar plate a circulating air stream was passed over the agar plate cultures and then through a charcoal filter (Chromtech GmbH, Idstein, Germany, Precision Char Coal Filter 5 mg) for 18–24 h. The absorbed volatiles were eluted with analytically pure dichloromethane (40–50 μL) and the extract (1 μL) was immediately analyzed by GC–MS. The rest was stored at −80 °C for future reference.

**GC–MS.** GC–MS analyses were carried out on an Agilent 7890A connected with an Agilent 5975C inert mass detector fitted with a HP5-MS fused silica capillary column (30 m, 0.25 mm i. d., 0.25 μm film, Agilent). Instrumental parameters were (1) inlet pressure 77.1 kPa, He 23.3 mL min^−1^, (2) injection volume 1.5 μL, (3) transfer line 300 °C, and (4) electron energy 70 eV. The operation mode was splitless (60 s valve time) and the carrier gas was He at 1.2 mL min^−1^. The GC was programmed as follows: 5 min at 50 °C increasing with 5 °C min^−1^ to 320 °C. Retention indices (*I*) were determined from a homologous series of *n*-alkanes (C_8_–C_38_).
